# Small Aperture Antenna Arrays for Direction of Arrival Estimation

**DOI:** 10.3390/s25123606

**Published:** 2025-06-08

**Authors:** Krutant J. Mehta, Inder J. Gupta

**Affiliations:** ElectroScience Laboratory, The Ohio State University, Columbus, OH 43212, USA; gupta.11@osu.edu

**Keywords:** small antenna arrays, direction-of-arrival estimation, array spatial covariance matrix, mutual coupling

## Abstract

In this paper, we establish criteria for the design of small aperture antenna arrays for Direction of Arrival (DOA) estimation. We define a small aperture antenna array as one consisting of a few elements with an average interelement spacing less than or equal to half a wavelength. We use the spatial covariance matrix of the antenna array to derive the design criterion. It is well known that the DOA estimation performance of an antenna array is strongly related to the amount of information in this matrix. Also, the Cramer-Rao Bound of the estimated DOA is closely related to this matrix. We establish and demonstrate that, for optimal DOA estimation performance, a small aperture antenna array should have non-uniformly spaced and dissimilar antenna elements. Since mutual coupling between antenna elements makes their in situ responses dissimilar, instead of mitigating mutual coupling, one should include mutual coupling in the DOA estimation process to enhance the DOA estimation performance of antenna arrays.

## 1. Introduction

Direction of Arrival (DOA) estimation of radio-frequency signals is used extensively in radar [1], navigation [2], and communication [3,4] systems. A crucial component of DOA estimation systems is the antenna array used to sample the incident signals. In the open literature, there has been only limited work on the design of antenna arrays for DOA estimation applications. Work in this area has mostly been restricted to the use of uniformly spaced antenna arrays with various linear and planar geometries. To satisfy the Shannon–Nyquist sampling criteria [5,6], an interelement spacing of half a wavelength between adjacent elements is used. In these arrays, due to small interelement spacing, there is some coupling between the antenna elements. In the DOA estimation literature, the focus has been on developing methods to mitigate or compensate for the mutual coupling effects in an antenna array [7,8,9,10,11]. In this paper, we show that, contrary to previous studies, mutual coupling between antenna array elements helps improve the DOA estimation capability of an antenna array. We also establish that non-uniform spacing between array elements similarly enhances the DOA estimation capability.

In our investigation, we use the Array Spatial Covariance Matrix (ASCM) to establish the desired properties of antenna arrays for DOA estimation. This matrix contains the pairwise cross-correlations between the samples collected at each antenna element. It is well known [12] that the performance of a DOA estimation system is strongly related to the amount of information in the ASCM. The Cramér–Rao Bound (CRB) [13,14,15,16] of the estimated signal directions is also strongly related to the ASCM. We show that when multiple signals are incident, the ASCM is the superposition of the covariance matrices corresponding to individual incident signals. In addition, the individual covariance matrices are simply the weighted outer products of the array response in the directions of one or two incident signals. The response and design of the antenna array, therefore, are of great importance for DOA estimation. We show that when the response of the antenna array is diverse and unstructured, the resulting ASCM contains more information about the incident signals.

There are several ways to make the array response diverse and unstructured. Antenna arrays with uniform spacing result in a structured response, which is not desirable. Instead, one can arrange the antenna elements non-uniformly. We show that non-uniform interelement spacings can increase the information in the ASCM. Another way to make the array response diverse is to use antenna elements with dissimilar radiation patterns. The use of different types of antenna elements can improve DOA estimation performance even for uniformly spaced arrays. However, it may not be practical to use different types of antenna elements in an array. In non-uniformly spaced antenna arrays, some elements may be closely spaced. These closely spaced elements will exhibit strong mutual coupling effects, which alter the in situ radiation patterns of individual elements, making them dissimilar. When mutual coupling is accounted for in the DOA estimation process and in the ASCM computation, we show that it can improve the DOA estimation performance of an antenna array.

To demonstrate the performance improvements due to non-uniform interelement spacing and mutual coupling, we study three different kinds of antenna arrays: linear, cross, and square geometries. For each geometry, we study an array with uniform spacing and another with non-uniform spacing, both with the same aperture. For each antenna array, we generate the array response with and without mutual coupling. This response is then used to compute the ASCM for various signal scenarios. To evaluate the performance of three different antenna arrays, we use a metric based on the well-known Cramér–Rao Bound (CRB), a popular tool for benchmarking the performance of DOA estimation systems. We show that non-uniformly spaced arrays that include mutual coupling in the array response exhibit better DOA estimation performance than uniformly spaced arrays without mutual coupling. Note that for all antenna arrays, the same response (with or without mutual coupling) is used both to compute the ASCM and in the DOA estimation process. Thus, there is no estimation error due to mismatch between the true in situ antenna response and the one used for DOA estimation.

Recently, thinned antenna arrays have been used for DOA estimation. In thinned arrays, elements that do not significantly contribute to array performance are removed to reduce system cost. These arrays use integer multiples of half-wavelength interelement spacing in a non-uniform fashion. Several notable thinned array designs have been proposed for DOA estimation. In Minimum Redundancy Arrays (MRA) [17], a search is performed to find an array configuration with the fewest repeated interelement spacings. In Nested Arrays (NA) [18], several uniform linear sub-arrays, each with different numbers of elements and interelement spacings, are combined. This results in a composite array with many unique interelement spacings and few redundancies. Co-Prime Arrays (CPA) [19] use co-prime numbers *N* and *M* to design an array with N+M−1 elements that also has only few redundant interelement spacings. Several extensions to these methods have been proposed in the literature [20,21,22,23,24]. However, all thinned arrays have an average interelement spacing that can be much greater than half a wavelength. As a result, the aperture for these arrays can be quite large when compared to a uniformly spaced array with half-wavelength spacing and the same number of antenna elements. Many applications simply do not have the area available to accommodate such large aperture antenna arrays. In this paper, we limit our discussion to antenna arrays with average interelement spacing of half a wavelength. Also, antenna arrays with few elements (less than ten) are used in our investigation. We call these antenna arrays as “Small Aperture Antenna Arrays”.

The remainder of this paper is organized as follows. In Section 2, we describe the narrowband signal model and provide the equations for the ASCM. We show how the ASCM is a superposition of individual covariance matrices and how it depends on the array response. Section 3 describes the response of antenna arrays with different spacings and the inclusion of mutual coupling in the array response. We also derive the corresponding array covariance matrix and show how its information content increases. In Section 4, we describe the CRB-based metric used to compare the DOA estimation performance of different arrays. The example antenna arrays and related signal scenarios are given in Section 5. We present numerical results using the CRB-based metric for the various antenna arrays in Section 6. Finally, Section 7 concludes the paper.

## 2. Signal Model and the Array Spatial Covariance Matrix

Consider an antenna array of *L* elements, and let *K* narrowband signals be incident on the array. The sources transmitting these signals are assumed to be stationary and located in the far field of the array. Let the incident signals overlap in both time and frequency. For simplicity, we assume that the signals and the antenna elements have a single, matching polarization. The signals are assumed to be Wide Sense Stationary (WSS). Finally, let the directions of the incident signals be unique and denoted by $\psi \in R^{K\times 1}$. Each direction $\psi_{k}$ for k=1,2,…,K corresponds to an elevation angle $\theta_{k}$ and an azimuth angle $\phi_{k}$. Then, the digitized samples received by various antenna elements are given by

(1) x(n)=A(ψ)s(n)+z(n),n=1,2,…,N,

where $x\left(n\right)\in C^{L\times 1}$ is the *n*th sample of the array output, $A\left(\psi \right)\in C^{L\times K}=\left[a\left(\psi_{1}\right),a\left(\psi_{2}\right),\ldots ,a\left(\psi_{K}\right)\right]$ is the matrix containing the array response in the directions of the incident signals, $s\left(n\right)\in C^{K\times 1}$ is the *n*th sample of the incident signals, and $z\left(n\right)\in C^{L\times 1}$ is the additive noise in the *n*th sample. A total of *N* samples are collected. Note that $a(\psi_{k})$ is the Array Response Vector (ARV), which contains the gain and phase response of the array in the direction $\psi_{k}$.

In the literature [12,14], a common assumption is that the signals follow a zero-mean, complex circular Gaussian random process with signal covariance matrix $P\in C^{K\times K}=E\left[s\left(n\right)s^{H}\left(n\right)\right]$. The noise is also assumed to be a zero-mean, complex circular Gaussian random process, with noise covariance matrix $Z\in C^{L\times L}=E\left[z\left(n\right)z^{H}\left(n\right)\right]$. We assume that the noise at each antenna element is uncorrelated with the noise at other elements and with the incident signals. The noise at each antenna element has the same variance, denoted by $\sigma^{2}$. Thus, the noise covariance matrix is diagonal and can be written as $Z=\sigma^{2}I_{L}$, where $I_{L}$ is the *L*-sized identity matrix.

DOA estimation is typically performed using the sample covariance matrix [12]. This is formed by the outer product of the sample vector and its conjugate transpose, averaged over all *N* signal samples. The sample covariance matrix is preferred for DOA estimation because it provides robustness to noise and reduces data dimensionality. Algorithms that use the sample covariance matrix are called Second-order-Statistics (SOS)-based DOA estimation methods. The sample covariance matrix is given by

(2) $$\hat{R}=\frac{1}{N}\sum_{n=1}^{N}x\left(n\right)x^{H}\left(n\right).$$

Note that $x^{H}\left(n\right)$ represents the conjugate transpose of x(n). The sample covariance matrix $\hat{R}\in C^{L\times L}$ is a square, Hermitian matrix. It contains all measurements of the incident signals and hence all available information. To understand the structure of the sample covariance matrix, we can expand (2) using (1), resulting in

(3) $$\begin{array}{cc} \hat{R}=\frac{1}{N}\sum_{n=1}^{N} & \left(A\left(\psi \right)s\left(n\right)+z\left(n\right)\right){\left(A\left(\psi \right)s\left(n\right)+z\left(n\right)\right)}^{H}\\ =\frac{1}{N}\sum_{n=1}^{N} & (A\left(\psi \right)s\left(n\right)s^{H}\left(n\right)A^{H}\left(\psi \right)\\ \; & +A\left(\psi \right)s\left(n\right)z^{H}\left(n\right)+z\left(n\right)s^{H}\left(n\right)A^{H}\left(\psi \right)\\ \; & +z\left(n\right)z^{H}\left(n\right)). \end{array}$$

From (3), we observe that the sample covariance matrix consists of four terms. The first term, $A\left(\psi \right)s\left(n\right)s^{H}\left(n\right)A^{H}\left(\psi \right)$, represents the noiseless covariance matrix and contains useful information about the signals. The last term, $z\left(n\right)z^{H}\left(n\right)$, represents only the noise. The cross-terms $A\left(\psi \right)s\left(n\right)z^{H}\left(n\right)$ and $z\left(n\right)s^{H}\left(n\right)A^{H}\left(\psi \right)$ vanish for a sufficiently large number of samples due to the assumed uncorrelated nature of signals and noise. That is, $E\left[z\left(n\right)s^{H}\left(n\right)\right]=E\left[s\left(n\right)z^{H}\left(n\right)\right]=0$. Assuming a large number of samples, the sample covariance matrix can be written as

(4) $$R=A\left(\psi \right)PA^{H}\left(\psi \right)+Z,$$

where $R\in C^{L\times L}=E\left[\hat{R}\right]=E\left[x\left(n\right)x^{H}\left(n\right)\right]$ is referred to as the Array Spatial Covariance Matrix (ASCM). In this form, the dependence of the sample covariance matrix on the array geometry, the signal properties, and the noise is clear. In (4), P is the incident signal correlation matrix. When the signals are correlated, all entries of P may be non-zero. If the incident signals are uncorrelated, the signal correlation matrix P is diagonal, meaning its off-diagonal entries are zero. DOA estimation is more challenging when signals are correlated [25,26].

Assuming correlated signals, there are up to $K+K^{2}+1$ unknowns in the DOA estimation problem: *K* unknown signal directions, $K^{2}$ entries of P, and the noise variance $\sigma^{2}$. The goal of DOA estimation is to determine the signal directions despite these unknowns. The ASCM is the only available observation for this estimation, and its information content is critical. Since R has $L^{2}$ entries, it potentially offers $L^{2}$ independent observations. Thus, maximizing the number of independent entries in the ASCM is crucial for robust DOA estimation, and this is closely tied to the design of the antenna array. To highlight this structure, we rewrite (4) as a double summation:

(5) $$\begin{array}{cc} R= & \sum_{u=1}^{K}\sum_{v=1}^{K}\left(p_{u,v}a\left(\psi_{u}\right)a^{H}\left(\psi_{v}\right)\right)+Z\\ = & \sum_{u=1}^{K}\sum_{v=1}^{K}\left(p_{u,v}\tilde{R}\left(\psi_{u},\psi_{v}\right)\right)+Z. \end{array}$$

In (5), we define a new quantity, $\tilde{R}\left(\psi_{u},\psi_{v}\right)$, called the Array Covariance Matrix (ACM). It is the outer product of the ARV in direction $\psi_{u}$ and the conjugate transpose of the ARV in direction $\psi_{v}$, for u,v∈{1,…,K}. The ACM depends only on the signal directions and the array geometry, making it useful for analyzing antenna array properties that impact DOA estimation. In the next section, we study the ARV of various antenna arrays and identify desirable array characteristics that help maximize the number of independent entries in the ACM. The greater the number of independent entries in the ACM, the more informative the ASCM becomes. To simplify the analysis, we will examine only the diagonal ACM terms $\tilde{R}\left(\psi_{u},\psi_{u}\right)$.

## 3. Array Response Vector

### 3.1. Linear Antenna Arrays

The design of the antenna array significantly impacts the DOA estimation performance of a system. To understand how the array design influences the information content of the ACM, let us investigate the well-known Uniform Linear Array (ULA). The ULA is commonly used for DOA estimation and has been frequently referenced in the open literature [27]. We will show that this array does not have the maximum number of independent entries in the ACM and does not offer optimal DOA estimation performance.

Consider an *L*-element ULA with similar antenna elements and an interelement spacing of d∈R. The first element is placed at the coordinate origin, with the remaining elements located along the positive x-axis. Let $f(\theta_{u},\phi_{u})\in C$ denote the response of an antenna element in the direction of the *u*th incident signal. This response is measured in free space when the antenna element is placed at the coordinate phase center. Recall that the direction of the *u*th incident signal is denoted by $\psi_{u}$, which corresponds to a pair of elevation and azimuth angles $\left(\theta_{u},\phi_{u}\right)$. Initially we will assume that there is no coupling between antenna elements. Then, the *ℓ*th entry of the ARV of a ULA in the $\left(\theta_{u},\phi_{u}\right)$ direction is given by

(6) $$\begin{array}{cc} a_{\ell }\left(\theta_{u},\phi_{u}\right)=f\left(\theta_{u},\phi_{u}\right) & e^{j\frac{2\pi }{\lambda }d\left(\ell -1\right)sin\theta_{u}cos\phi_{u}},\\ \; & \;\ell =1,2,\ldots ,L, \end{array}$$

where *j* is the complex unit and λ is the wavelength of operation. The exponential term is independent of the type of antenna element used and represents the phase shift caused by the displacement of an antenna element from the phase center of the array.

Using this expression for the ARV, the corresponding ACM is given by

(7) $$\begin{array}{cc} \tilde{R}\left(\psi_{u},\psi_{u}\right) & =\\ \; & f\left(\theta_{u},\phi_{u}\right)f^{*}\left(\theta_{u},\phi_{u}\right)e^{j\frac{2\pi }{\lambda }D_{U}sin\theta_{u}cos\phi_{u}}, \end{array}$$

where $f^{*}\left(\theta_{u},\phi_{u}\right)$ is the conjugate of $f(\theta_{u},\phi_{u})$. Note this equation comprises of an exponential term with a matrix in the exponent. This notation indicates element-by-element processing over each entry in the matrix. The matrix $D_{U}\in R^{L\times L}$ is called the difference co-array matrix and is given by

(8) $$D_{U}=\left[\begin{array}{cccc} 0 & -d & \ldots & -(L-1)d\\ d & 0 & \ldots & -(L-2)d\\ 2d & d & \ldots & -(L-3)d\\ \vdots & \vdots & \; & \vdots \\ (L-1)d & (L-2)d & \ldots & 0 \end{array}\right].$$

Based on (7) and (8), all values of $\tilde{R}\left(\psi_{u},\psi_{u}\right)$ share common factors and differ only in the entries of $D_{U}$. The entries of the difference co-array matrix depend only on the difference between element indices. As a result, $D_{U}$ has a Toeplitz structure where all diagonals contain the same value. Consequently, the ACM for the ULA is also Toeplitz. Due to this structure, out of a total of $L^{2}$ entries in the ACM, only 2L−1 are independent; the remaining entries are redundant. This is why a ULA with similar antenna elements does not contain the maximum amount of information in its covariance matrix. Therefore, the ULA with similar antenna elements is limited in its DOA estimation capability, leaving room for improvement.

To enhance the performance of the linear array, one can use non-uniform spacing. This has been previously studied in the thinned array literature. Let $\bar{x}\in R^{L\times 1}$ denote the positions of the elements of a Non-Uniform Linear Array (NULA) with arbitrary non-uniform spacing. Then, the *ℓ*th entry of the ARV for the NULA in the direction $\left(\theta_{u},\phi_{u}\right)$ is given by

(9) $$\begin{array}{cc} a_{\ell }\left(\theta_{u},\phi_{u}\right)=f\left(\theta_{u},\phi_{u}\right) & e^{j\frac{2\pi }{\lambda }{\bar{x}}_{\ell }sin\theta_{u}cos\phi_{u}},\\ \; & \;\ell =1,2,\ldots ,L. \end{array}$$

We observe that the ARV now depends on the position of the *ℓ*th antenna element rather than just the index. Using this definition for the ARV, the equation for the ACM for the NULA can be written as

(10) $$\begin{array}{cc} \tilde{R}\left(\psi_{u},\psi_{u}\right) & =\\ \; & f\left(\theta_{u},\phi_{u}\right)f^{*}\left(\theta_{u},\phi_{u}\right)e^{j\frac{2\pi }{\lambda }D_{NU}sin\theta_{u}cos\phi_{u}} \end{array}$$

where $\tilde{R}\left(\psi_{u},\psi_{u}\right)\in C^{L\times L}$ is the ACM, and $D_{NU}\in R^{L\times L}$ is the difference co-array matrix given by

(11) $$D_{NU}=\left[\begin{array}{cccc} 0 & -({\bar{x}}_{2}-{\bar{x}}_{1}) & \ldots & -({\bar{x}}_{L}-{\bar{x}}_{1})\\ \left({\bar{x}}_{2}-{\bar{x}}_{1}\right) & 0 & \ldots & -({\bar{x}}_{L}-{\bar{x}}_{2})\\ \left({\bar{x}}_{3}-{\bar{x}}_{1}\right) & \left({\bar{x}}_{3}-{\bar{x}}_{2}\right) & \ldots & -({\bar{x}}_{L}-{\bar{x}}_{3})\\ \vdots & \vdots & \; & \vdots \\ \left({\bar{x}}_{L}-{\bar{x}}_{1}\right) & \left({\bar{x}}_{L}-{\bar{x}}_{2}\right) & \ldots & 0 \end{array}\right].$$

We see that due to the non-uniform spacing, the difference co-array matrix for the NULA in (11) is no longer Toeplitz. Different off-diagonal terms, such as $\left({\bar{x}}_{2}-{\bar{x}}_{1}\right)$ and $\left({\bar{x}}_{3}-{\bar{x}}_{2}\right)$, can have independent values. Because the difference co-array matrix is no longer Toeplitz, the ACM also loses its Toeplitz structure. This allows more entries of the ACM to be independent. In fact, for a properly designed NULA, all the off-diagonal entries can be independent. Such an array can have up to $L^{2}-L+1$ independent entries. This increase in the number of independent entries should lead to improved DOA estimation performance. However, the diagonal entries of the difference co-array matrix are still zero, so the ACM retains some redundancy.

### 3.2. Planar Arrays

In addition to non-uniform spacing in a linear array, one can arrange the antenna elements in two dimensions. Arrays of this type are called “planar” arrays. Planar arrays offer several advantages. The additional dimension allows planar arrays to receive signals from the entire 360° azimuthal plane. Furthermore, we find that even planar arrays with uniformly spaced elements can have more independent entries in the ACM than the ULA. Let the elements of the planar array be placed in the x-y plane, and let $\bar{x}\in R^{L\times 1}$ and $\bar{y}\in R^{L\times 1}$ denote the positions of the antenna elements. Note that all antenna elements in the array still have similar radiation patterns, denoted by $f(\theta_{u},\phi_{u})$. Then, the *ℓ*th entry of the ARV for a planar array, in the direction of the *u*th signal, is given by

(12) $$\begin{array}{cc} a_{\ell }\left(\theta_{u},\phi_{u}\right)=f\left(\theta_{u},\phi_{u}\right) & e^{j\frac{2\pi }{\lambda }sin\theta_{u}\left({\bar{x}}_{\ell }cos\phi_{u}+{\bar{y}}_{\ell }sin\phi_{u}\right)},\\ \; & \;\ell =1,2,\ldots ,L. \end{array}$$

We see that the ARV of the planar array depends on both the *x* and *y* positions of each antenna element. The ACM for a planar array is given by

(13) $$\begin{array}{cc} \tilde{R}\left(\psi_{u},\psi_{u}\right) & =\\ f(\theta_{u},\phi_{u}) & f^{*}\left(\theta_{u},\phi_{u}\right)e^{j\frac{2\pi }{\lambda }sin\theta_{u}\left(D_{x}cos\phi_{u}+D_{y}sin\phi_{u}\right)} \end{array}$$

where there are now two separate difference co-array matrices for each dimension. They are given by

(14) $$D_{x}=\left[\begin{array}{cccc} 0 & -({\bar{x}}_{2}-{\bar{x}}_{1}) & \ldots & -({\bar{x}}_{L}-{\bar{x}}_{1})\\ \left({\bar{x}}_{2}-{\bar{x}}_{1}\right) & 0 & \ldots & -({\bar{x}}_{L}-{\bar{x}}_{2})\\ \left({\bar{x}}_{3}-{\bar{x}}_{1}\right) & \left({\bar{x}}_{3}-{\bar{x}}_{2}\right) & \ldots & -({\bar{x}}_{L}-{\bar{x}}_{3})\\ \vdots & \vdots & \; & \vdots \\ \left({\bar{x}}_{L}-{\bar{x}}_{1}\right) & \left({\bar{x}}_{L}-{\bar{x}}_{2}\right) & \ldots & 0 \end{array}\right],$$

(15) $$D_{y}=\left[\begin{array}{cccc} 0 & -({\bar{y}}_{2}-{\bar{y}}_{1}) & \ldots & -({\bar{y}}_{L}-{\bar{y}}_{1})\\ \left({\bar{y}}_{2}-{\bar{y}}_{1}\right) & 0 & \ldots & -({\bar{y}}_{L}-{\bar{y}}_{2})\\ \left({\bar{y}}_{3}-{\bar{y}}_{1}\right) & \left({\bar{y}}_{3}-{\bar{y}}_{2}\right) & \ldots & -({\bar{y}}_{L}-{\bar{y}}_{3})\\ \vdots & \vdots & \; & \vdots \\ \left({\bar{y}}_{L}-{\bar{y}}_{1}\right) & \left({\bar{y}}_{L}-{\bar{y}}_{2}\right) & \ldots & 0 \end{array}\right].$$

The ACM for planar arrays offers more flexibility than the ACM for linear arrays. We see that the independence of an entry depends on the difference in both the *x* and *y* positions of the corresponding antenna elements. Additionally, the difference co-array entries are scaled by the sine and cosine of the azimuth angle. Due to this, there can be significant directional variability in the ACM. Planar antenna arrays can have up to $L^{2}-L+1$ independent entries in the ACM. We have shown that non-uniform geometry helps linear arrays—this is also true for planar arrays. Whenever the differences $\left({\bar{x}}_{p}-{\bar{x}}_{q}\right)$ and $\left({\bar{y}}_{p}-{\bar{y}}_{q}\right)$ for p,q∈[1,K],p≠q are the same, the corresponding ACM entries will match. This can happen if the planar array has any kind of uniform spacing. One should avoid this by designing the array with non-uniform spacing. Comparisons of DOA estimation performance between uniform and non-uniform planar arrays will be shown in Section 6. For both uniform and non-uniform planar arrays, the ACM still falls short of the maximum of $L^{2}$ independent entries in the covariance matrix because the diagonal entries will still be the same. We discuss how to achieve this in the next section.

### 3.3. Dissimilar Antenna Elements

In the analysis thus far, we have assumed that the antenna elements have similar radiation patterns. In doing so, we found that arrays with non-uniform interelement spacing can achieve up to $L^{2}-L+1$ independent entries in the ACM. This is the limit because the diagonal entries of the ACM will still be the same regardless of the array geometry. However, using different types of antenna elements can change this. Let all elements of an antenna array be dissimilar. Under this assumption, we rewrite the radiation pattern in the $\left(\theta_{u},\phi_{u}\right)$ direction as $f_{\ell }\left(\theta_{u},\phi_{u}\right)$, which is the response of the *ℓ*th antenna element when the element is placed in the phase center of the coordinate system. Now, the *ℓ*th term of the ARV in the $\left(\theta_{u},\phi_{u}\right)$ direction of a NULA will be given by

(16) $$\begin{array}{cc} a_{\ell }\left(\theta_{u},\phi_{u}\right)=f_{\ell }\left(\theta_{u},\phi_{u}\right) & e^{j\frac{2\pi }{\lambda }{\bar{x}}_{\ell }sin\theta_{u}cos\phi_{u}},\\ \; & \;\ell =1,2,\ldots ,L. \end{array}$$

Note that ${\bar{x}}_{\ell }$ represents the x coordinate of the *ℓ*th antenna element in the array.

Using this ARV, the ACM of the NULA in the $\left(\theta_{u},\phi_{u}\right)$ direction, using dissimilar antenna elements, is given by $\tilde{R}\left(\psi_{u},\psi_{u}\right)\in C^{L\times L}$ as follows

(17) $$\tilde{R}\left(\psi_{u},\psi_{u}\right)=F⊙e^{j\frac{2\pi }{\lambda }D_{NU}sin\theta_{u}cos\phi_{u}}$$

This equation includes a Hadamard product denoted by ⊙, which represents the element-by-element matrix product. The difference co-array matrix $D_{NU}$ for the NULA is the same as in (11), and the array response matrix F is defined as

(18) $$F=\left[\begin{array}{cccc} f_{1}f_{1}^{*} & f_{1}f_{2}^{*} & \ldots & f_{1}f_{L}^{*}\\ f_{2}f_{1}^{*} & f_{2}f_{2}^{*} & \ldots & f_{2}f_{L}^{*}\\ f_{3}f_{1}^{*} & f_{3}f_{2}^{*} & \ldots & f_{3}f_{L}^{*}\\ \vdots & \vdots & \; & \vdots \\ f_{L}f_{1}^{*} & f_{L}f_{2}^{*} & \ldots & f_{L}f_{L}^{*} \end{array}\right].$$

When the antenna elements are dissimilar, the difference co-array matrix remains the same, but the antenna radiation pattern matrix $F\in C^{L\times L}$ is a new term. This matrix can potentially have $L^{2}$ independent entries depending on the radiation pattern of each antenna element. Due to this, the maximum number of independent entries in the ACM can be $L^{2}$. In this way, even antenna arrays with uniform spacing can achieve the maximum of $L^{2}$ independent entries in the ACM.

In real antenna arrays, one does not have to use different types of antenna to induce dissimilarity in the array response. Closely spaced antenna elements in an array will exhibit strong Mutual Coupling (MC) effects. This is especially true for arrays with fixed aperture and non-uniform spacing, as some antenna elements can be even closer together. Often in array signal processing, MC in antenna arrays is seen as a nuisance factor and antenna arrays are designed to mitigate any coupling between antenna elements. Mutual coupling between antenna elements would change the response of even similar antenna elements. This, in turn, should lead to more independent entries in the ACM and improved DOA estimation performance. Instead of trying to mitigate mutual coupling, one can simulate or measure the mutual coupling effects and include those in the DOA estimation process.

The ACM of an antenna array needs to have as many independent entries as possible in order to optimize DOA estimation. We have identified two factors in antenna arrays that can improve the number of independent entries in the ACM.

1. Antenna arrays should be designed with non-uniform interelement spacing. This is true for both linear and planar antenna arrays.
2. Instead of attempting to mitigate mutual coupling, one should include it in the response of an antenna array. We find that mutual coupling can actually increase the number of independent entries in the ACM of an antenna array, and therefore should improve DOA estimation.

In this paper, we will investigate the DOA estimation performance of several uniform and non-uniform antenna arrays. To quantify the antenna array performance, we will use a metric based on the well known Cramer–Rao Bound for DOA estimation. The performance metric is discussed next.

## 4. Performance Metric

The Cramer–Rao Bound (CRB) [28] is a commonly used tool for the analysis of statistical estimation systems. In general, the CRB gives the minimum variance of an unbiased estimator of unknown parameters of a random variable. For DOA estimation, one can use it to find the minimum estimation variance of the unknown signal directions [13,14,15,16]. This information can be used to compare different antenna arrays for their DOA estimation performance.

The CRB is defined in terms of the unknown parameters of a random variable. Previously, we discussed how the DOA estimation system can have up to $q=K+K^{2}+1$ unknown variables. Let these variables be arranged in a vector $\alpha =[\psi ,p,\sigma^{2}]$. Here, $p\in C^{K^{2}\times 1}=vec\left(P\right)$ is the vectorized form of the signal covariance matrix, and the vec() function stacks the columns of a matrix into a single vector. Then, the CRB matrix is defined as the inverse of the Fisher Information Matrix (FIM). Let $J\left(\alpha \right)\in C^{q\times q}$ denote the FIM as a function of the vector of unknowns α. Under the Gaussian assumption of the signals and noise, the element in the *u*th row and *v*th column of the FIM is given by

(19) $$J_{u,v}\left(\alpha \right)=Ntr\left(R^{-1}\frac{\partial R}{\partial \alpha_{u}}R^{-1}\frac{\partial R}{\partial \alpha_{v}}\right),$$

where R is the ASCM given in (4). Then, the CRB matrix, denoted by $B\left(\alpha \right)\in C^{q\times q}$, is defined as the inverse of the FIM. In other words,

(20) $$B\left(\alpha \right)=J^{-1}\left(\alpha \right).$$

In (20), the inverse of the FIM is simple to compute numerically. However, in computing the FIM, the partial derivatives of each unknown variable require some simplification. We now focus our derivation on the partial derivatives $\frac{\partial R}{\partial \alpha_{u}}$ for all *u*. We note that there are three different types of unknown variables; the DOA’s, the entries of the signal covariance matrix, and the noise entries. Therefore, the partial derivatives can also be split into three different equations: $\frac{\partial R}{\partial \psi_{k}},\frac{\partial R}{\partial p_{u,v}}$ and $\frac{\partial R}{\partial \sigma^{2}}$. The form of these partial derivatives has been studied before in [15,16], so we list only the final forms.

We will start with the derivative of R with respect to the *k*th unknown signal direction, $\psi_{k}$. It can be shown that the derivative has the following form:

(21) $$\begin{array}{cc} \frac{\partial R}{\partial \psi_{k}}=\sum_{u=1}^{K}p_{k,u}\dot{a}\left(\psi_{k}\right)a^{H}\left(\psi_{u}\right) & +p_{u,k}a\left(\psi_{u}\right){\dot{a}}^{H}\left(\psi_{k}\right),\\ \; & \;k=1,2,\ldots ,K \end{array}$$

where $\dot{a}\left(\psi_{k}\right)=\frac{\partial a(\psi_{k})}{\partial \psi_{k}}$ is the partial derivative of the ARV from the *k*th incident signal direction, and $p_{u,k}$ is the entry in the *u*th row and *k*th column of P. Note that the above form is generalized for correlated and uncorrelated signals. When the signals are uncorrelated, $p_{u,k}=0$ where u≠k and the summation collapses to a single term.

Next, the partial derivative of R with respect to the entry in the *u*th column and *v*th row in P matrix, $p_{u,v}$, is given by the outer product of the ARV for the *u*th incident signal and the conjugate transpose of the ARV for the *v*th incident signal

(22) $$\frac{\partial R}{\partial p_{u,v}}=a\left(\psi_{u}\right)a^{H}\left(\psi_{v}\right),\;u,v\in \left[1,K\right].$$

Note that this is simply the ACM, $\tilde{R}\left(\psi_{u},\psi_{v}\right)$.

Finally, the partial derivative of the ASCM R with respect to the *ℓ*th noise variance is given by

(23) $$\frac{\partial R}{\partial \sigma^{2}}=I_{L},$$

where $I_{L}$ is the *L*-sized identity matrix as before.

Most of these partial derivatives are based on the ARV in the incident signal directions. The ARV and its derivative are then of great importance in computing the CRB for DOA estimation. For real antenna arrays, the ARV is not available in a closed form, and one typically only has access to discrete measurements of the gain and phase of the antenna elements in various directions. In order to compute the derivative of the ARV, one must resort to numerical differentiation. There are many available methods to obtain the numerical derivative of discrete data, and we use the central differencing method as follows to compute the numerical derivative of the ARV for the *k*th signal $\psi_{k}$

(24) $$\frac{\partial a(\psi_{k})}{\partial \psi_{k}}\approx \frac{a\left(\psi_{k}+\Delta_{\psi }\right)-a\left(\psi_{k}-\Delta_{\psi }\right)}{2\Delta_{\psi }},$$

where $\Delta_{\psi }$ is a small angular shift (such as 0.01°).

Typically, one does not use all the values of the CRB matrix, only the first *K* diagonal entries that correspond to the minimum variance of the incident signal directions are of interest. Additionally, while the variance is useful, it is more intuitive to study the standard deviation because it has units degrees. Let $b\left(\alpha \right)\in R^{q\times 1}$ represent the square root of the diagonal entries of B(α). This is written as

(25) $$b\left(\alpha \right)=\sqrt{diag(B(\alpha ))}.$$

Then, one can decompose this vector into the CRB values for each type of variable $b\left(\alpha \right)=[b\left(\psi \right),b\left(p\right),b\left(\sigma^{2}\right)]$. Because we are interested in the CRB for the signal directions, we focus on b(ψ). However, we are more interested in a single quantity that explains the DOA estimation performance of all of the signals. For this, we take the average of b(ψ) over all *K* signals. This is defined as η∈R as follows

(26) $$\eta =\frac{1}{K}\sum_{i=1}^{K}b\left(\psi_{i}\right).$$

In (26), η represents the mean CRB for only a single set of incident signals. In order to compare antenna arrays thoroughly, we performed Monte Carlo simulations. In the results shown in this paper, for a given number of incident signals, we randomly vary the directions of the incident signals over *T* Monte Carlo simulations. For each simulation, we compute η. To study the average over all simulations, we take the mean of η over all trials, as in the following equation

(27) $$\tilde{\eta }=\frac{1}{T}\sum_{i=1}^{T}\eta \left(i\right).$$

We will refer to $\tilde{\eta }$ as the “Mean CRB” in the rest of this paper. Note that smaller values of the CRB correspond to better DOA estimation performance. This metric will be used to compare the DOA estimation performance of different antenna arrays. In the next section, we describe various antenna arrays used in our investigation.

## 5. Antenna Arrays

In our investigation, we have considered three types of antenna arrays. The first antenna array is a linear antenna array, the second is a cross antenna array, and the third is a square antenna array. For each geometry, we consider an array with uniform interelement spacing and an array with non-uniform interelement spacing. The non-uniform antenna array is not necessarily optimized—it was designed via trial and error. Both the uniform and non-uniform arrays have the same number of antenna elements and aperture. For all antenna arrays, nine half-wavelength dipole antenna elements are used. In addition, the antenna elements are placed in the x-y plane and the dipoles are oriented along the z-axis. For each array, we consider two cases for the ARV. In the first case, we do not compute the mutual coupling between antenna elements; i.e., the mutual coupling is ignored in the calculation of the ASCM and as well as in DOA estimation. This case will be referred to as “Without MC” in the results. On the other hand, the results marked “With MC” correspond to the array response that includes the mutual coupling between the antenna elements in the ASCM and in the DOA estimation process. The in situ ARV of the various antenna arrays over the angular region of interest was simulated using the Ansys HFSS electromagnetics simulation software. We note that in all cases, the same array response is used to generate the covariance matrix and compute the CRB-based metric; there is no mismatch.

**Figure 1:**
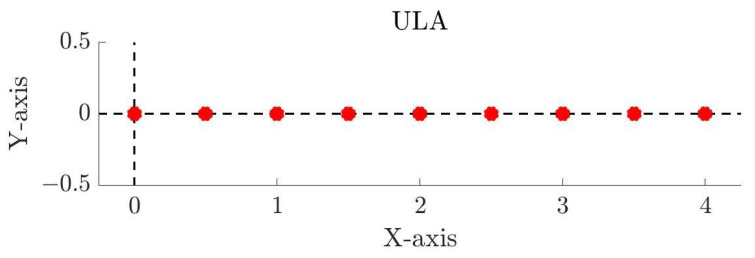
ULA with nine elements and half-wavelength interelement spacing. The aperture is 4λ, the axes are normalized to the wavelength, and the red circles mark the antenna elements.

Starting with the linear arrays, the ULA is designed so adjacent elements have half-wavelength interelement spacing. Since there are nine antenna elements, the total aperture is equal to 4λ. The first antenna element is placed on the coordinate origin and the remaining elements are placed on the positive x axis. The element distribution of this array is shown in Figure 1. For the NULA, the first and last antenna element are the same as the ULA, which keeps the aperture fixed. The remaining seven elements are repositioned so that the array has a non-uniform spacing. The positions of the antenna elements for both the ULA and the NULA are given in the first two columns of Table 1.

**Figure 2:**
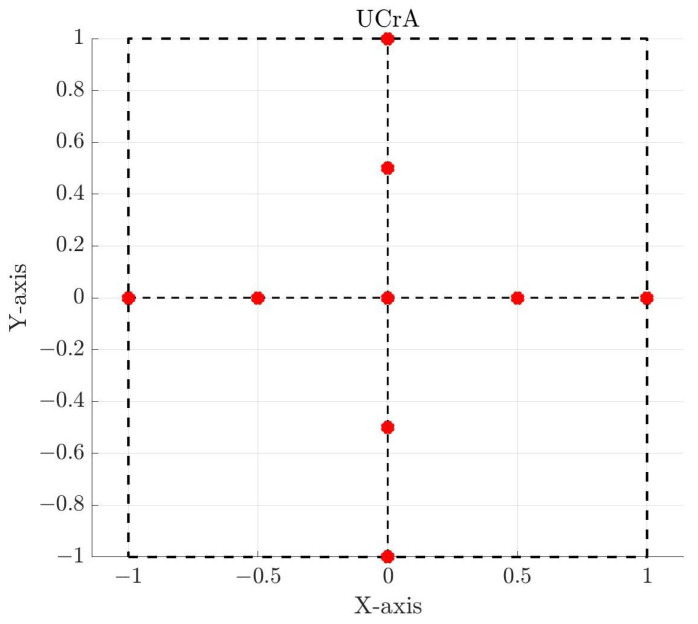
Uniform cross antenna array with nine elements and half-wavelength interelement spacing. The axes are normalized to the wavelength, and the red circles mark the antenna elements.

The Uniform Cross Array (UCrA) has an antenna element at the coordinate origin, and the remaining elements on the x and y axes with half-wavelength spacing between adjacent elements. Thus, the total aperture of the array is a square with side length 2λ. The element distribution of this geometry is shown in Figure 2. The Non-Uniform Cross Array (NUCrA) has non-uniform spacing. The center and edge antenna elements are still fixed to their positions, but the internal elements are moved. Table 1 lists the positions of the elements for these arrays, in the columns marked UCrA and NUCrA.

**Figure 3:**
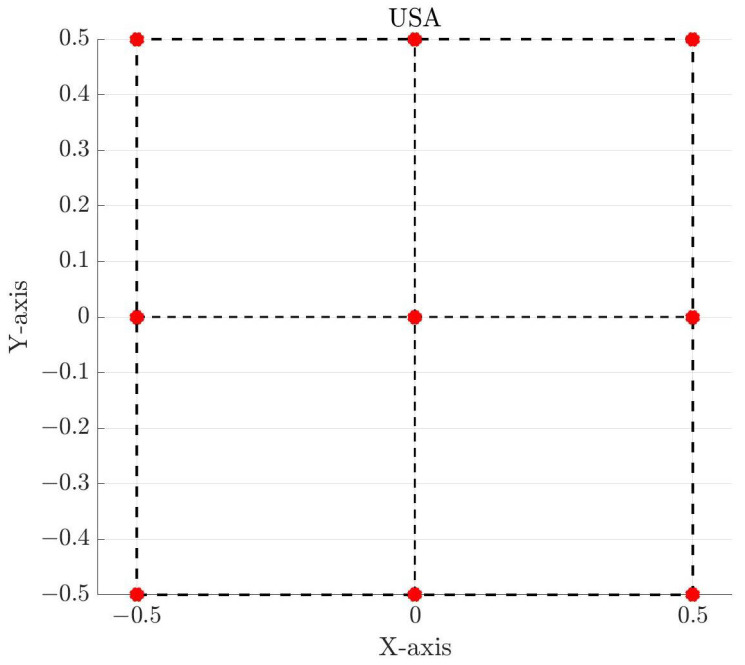
Uniform square antenna array with nine elements and half-wavelength interelement spacing. The axes are normalized to the wavelength, and the red circles mark the antenna elements.

Finally, the square arrays are characterized by antenna elements placed in a square aperture of size 1λ by 1λ, centered on the coordinate origin. For the Uniform Square Array (USA), the first antenna element is placed on the origin, an antenna element is placed on each of the four corners of the square, and the remaining elements are distributed on the edge of the square with half-wavelength interelement spacing. This geometry is shown in Figure 3. For the Non-Uniform Square Array (NUSA), the center element and four corner elements are held fixed, but the remaining elements are moved. The exact positions of the antenna elements for these arrays can be found in the last two columns of Table 1.

### Signal Scenarios

In all the simulations, we will have twelve or less signals incident. All signals will have equal power and the SNR for each incident signal is equal to 10 dB on an isotropic element. We will assume that 1000 samples are used in DOA estimation. We conduct twenty-five Monte Carlo simulations where the directions of the incident signals vary from one trial to the next. All signals are incident in the x-y plane, so the elevation angle θ=90°. For linear arrays, the incident signals in a given trial are randomly distributed in ϕ=[10°,170°], with a minimum spacing of 10° between adjacent signals. This is because linear arrays have poor resolution in the end-fire directions. Planar arrays are able to cover the entire azimuthal plane. For planar arrays, the incident signals are distributed randomly in ϕ=[0°,360°) and there is a minimum of 10° between adjacent signals. Additionally, in comparing the CRB in various cases, we need to select a performance requirement. Although this can differ based on the application, an accuracy of 0.1° is deemed appropriate. DOA estimation is only declared possible if the average of the mean CRB over all trials is below this threshold.

Finally, the correlation between the signals is varied and will be explained in more detail for each set of results in the following section. Recall that the correlation between the incident signals impacts the elements in the signal covariance matrix P. The general form of P when *K* correlated signals are incident is given by the following equation

(28) $$P=\left[\begin{array}{cccc} p_{1,1} & p_{1,2} & \ldots & p_{1,K}\\ p_{2,1} & p_{2,2} & \ldots & p_{2,K}\\ p_{3,1} & p_{3,2} & \ldots & p_{3,K}\\ \vdots & \vdots & \; & \vdots \\ p_{K,1} & p_{K,2} & \ldots & p_{K,K} \end{array}\right].$$

In (28), several quantities need to be defined in more detail. Each entry of the signal covariance matrix represents the expected value $p_{u,v}=E\left[s_{u}\left(n\right)s_{v}^{*}\left(n\right)\right]$ for u,v∈[1,K]. The diagonal entries $p_{u,u}$ for u=1,2,…,K represent the power of each of the *K* signals. Then, the off-diagonal entries represent the cross-correlation between the various incident signals. Note that the off-diagonal entries are given in terms of a correlation coefficient. This is expressed in the below equation:

(29) $$p_{u,v}=\omega_{u,v}\sqrt{p_{u,u}p_{v,v}},\;u,v\in \left[1,K\right],\;u\neq v$$

where $\omega_{u,v}\in C$ with $|\omega_{u,v}|<1$ is the correlation coefficient between the *u*th and *v*th signals and $p_{u,u}$ is the power of the *u*th signal, u=1,2,…,K. We assume that the signals are only partially correlated; therefore the correlation coefficient is less than unity. Note that the signal covariance matrix is Hermitian because the correlation coefficients $\omega_{u,v}$ and $\omega_{v,u}$ are complex conjugates. In our simulations, we assume that the correlation coefficient is the same for all off-diagonal entries of P, and this correlation coefficient is denoted by ω.

In the next section, we use numerical simulations to compare the DOA estimation performance of these arrays using the CRB-based metric.

## 6. Numerical Results

**Figure 4:**
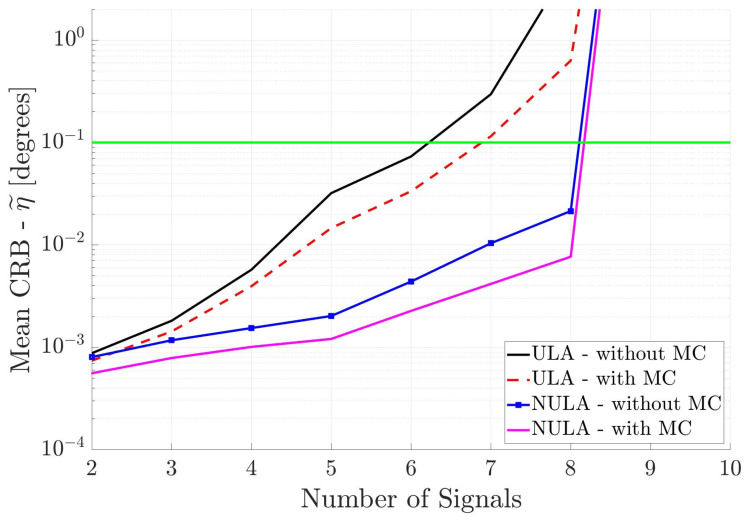
Mean CRB $\left(\tilde{\eta }\right)$ for linear arrays versus the number of incident signals. All incident signals are partially correlated.

In the first set of simulations, the incident signals are assumed to partially correlated with the correlation coefficient set to ω=0.5. This means that all entries of the signal covariance matrix P are nonzero. Thus, the number of unknowns in the DOA estimation system is $q=K+K^{2}+1$. We compute the mean CRB for the linear, cross and circular arrays. Figure 4 shows the performance of the two linear arrays, with and without MC, versus the number of incident signals. As the number of incident signals increases, then as expected, the mean CRB values increase. However, the different arrays have significantly different performance. The ULA without MC has the largest mean CRB values and therefore the poorest performance, crossing the 0.1° threshold a little after six signals. When mutual coupling is included in the array response, the mean CRB for the ULA with MC decreases slightly, crossing the threshold just before seven incident signals. The mean CRB for the NULA without MC is much lower than the mean CRB for the uniform array, and the NULA with MC has the lowest mean CRB curve indicating the best performance. The results show that mutual coupling and non-uniform spacing in the antenna array improve DOA estimation performance. There is a difference of several orders of magnitude in the mean CRB values from the ULA without MC to the NULA with MC. Interestingly, when there are more than eight signals incident, the mean CRB curves increase significantly for all arrays. This implies that underdetermined DOA estimation of partially correlated signals is not possible, regardless of the geometry or the response of the linear array. Although there are a total of twelve incident signals, the x-axis in Figure 4 only goes to ten signals due to this limit.

**Figure 5:**
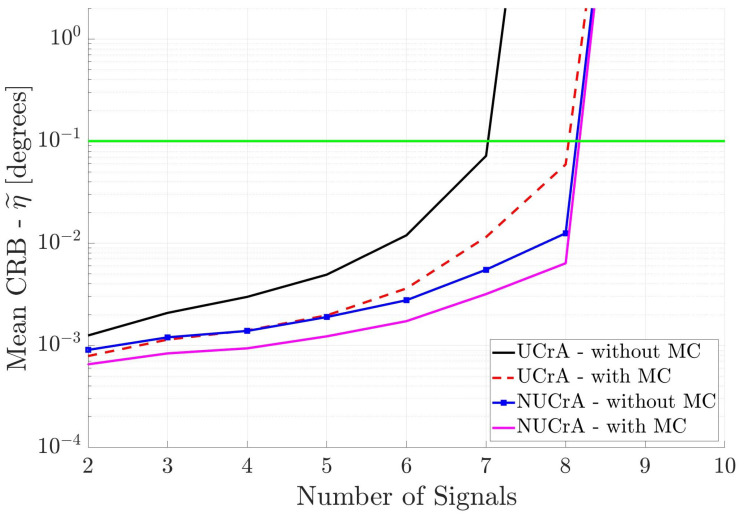
Mean CRB $\left(\tilde{\eta }\right)$ for cross arrays versus the number of incident signals. All incident signals are partially correlated.

Figure 5 shows the results for the two cross arrays when the incident signals are partially correlated. We see that the UCrA without MC has the poorest performance, whereas, the NUCrA with MC has the best performance. Again, the estimated DOA accuracy degrades as the number of signals increases. Also, none of the antenna arrays can estimate the DOA of more than eight incident signals.

**Figure 6:**
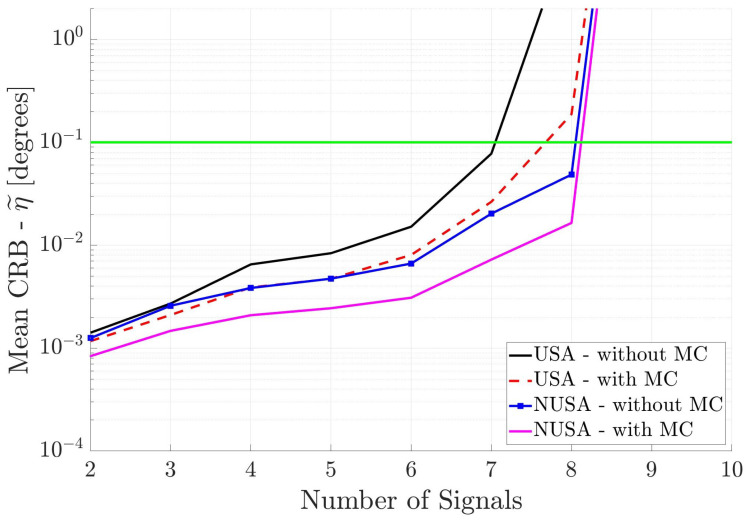
Mean CRB $\left(\tilde{\eta }\right)$ for square arrays versus the number of incident signals. All incident signals are partially correlated.

Finally, Figure 6 shows the performance of the two square arrays. Again, one can draw the same conclusions. Note that the NUSA with MC has the best performance.

Next, we will consider the case when the signals incident on the antenna arrays are uncorrelated from each other. As the signals are completely uncorrelated, the correlation coefficient for all cases is ω=0. Previously, we noted that DOA estimation for uncorrelated incident signals is easier than in the case of correlated incident signals. A subtle but important assumption is whether one is aware of the correlation between the signals, and whether this information is used in the DOA estimation process. We first assume that the signals are completely uncorrelated and that P is diagonal, but this information is not known. Therefore, there are still $q=K+K^{2}+1$ unknown parameters in the DOA estimation system. Exactly $K^{2}-K$ entries correspond to the off-diagonal entries of P and are zero, but this information is not available.

**Figure 7:**
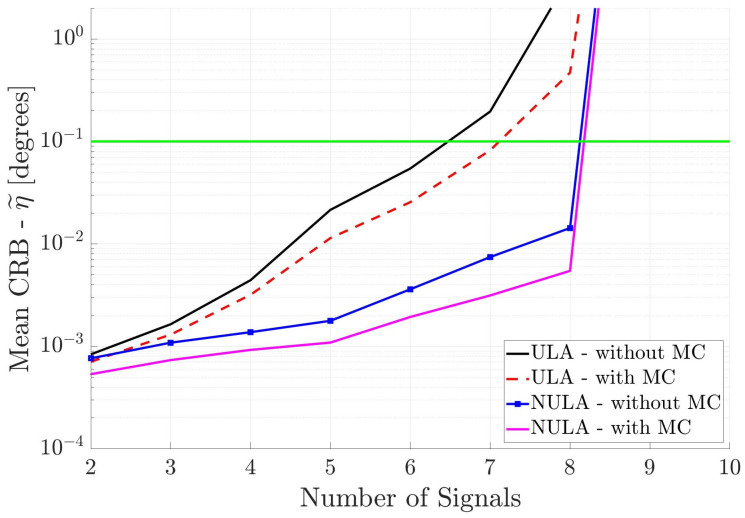
Mean CRB $\left(\tilde{\eta }\right)$ for linear arrays versus the number of incident signals. All incident signals are mutually uncorrelated, but this information is not used in DOA estimation.

**Figure 8:**
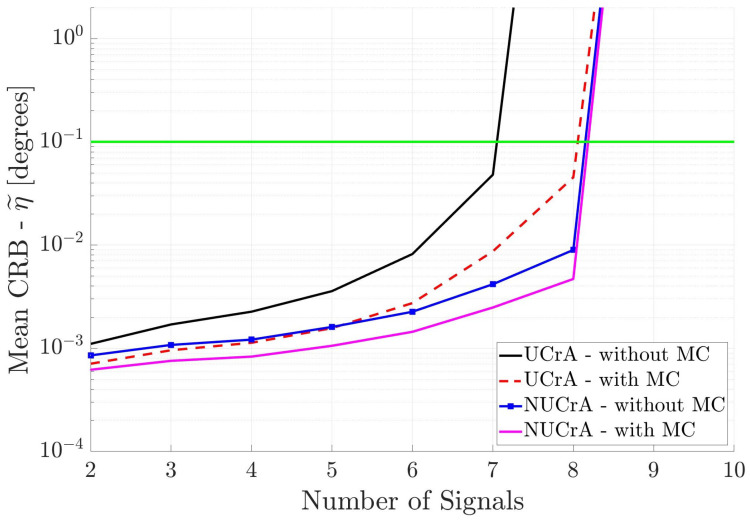
Mean CRB $\left(\tilde{\eta }\right)$ for cross arrays versus the number of incident signals. All incident signals are mutually uncorrelated, but this information is not used in DOA estimation.

**Figure 9:**
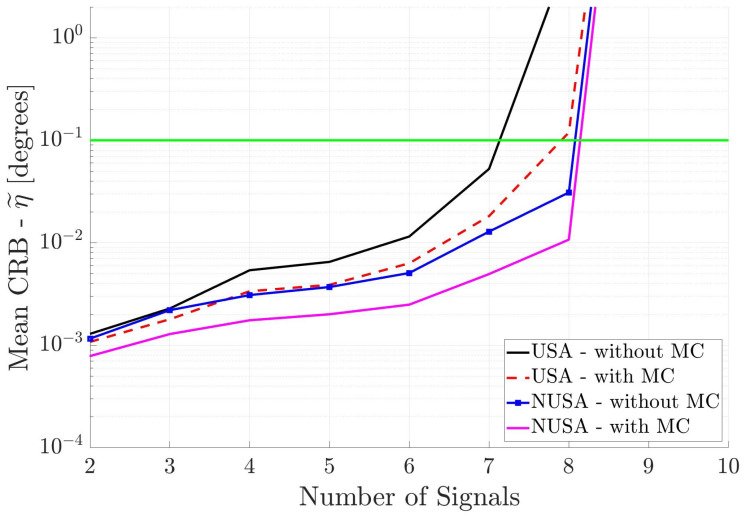
Mean CRB $\left(\tilde{\eta }\right)$ for square arrays versus the number of incident signals. All incident signals are mutually uncorrelated, but this information is not used in DOA estimation.

Figure 7, Figure 8, and Figure 9, respectively, show the DOA estimation performance of the linear antenna arrays, cross antenna arrays, and square antenna arrays when all incident signals are uncorrelated. Again, one can draw the same conclusions; i.e., non-uniformly spaced antenna arrays where the MC is included in the array response have the best performance.

If one compares the antenna array DOA estimation performance in Figure 7, Figure 8, and Figure 9 with that in Figure 4, Figure 5, and Figure 6, respectively, one will notice that the performance for the uncorrelated signals is only marginally better than for the partially correlated signals. The reason for this behavior is that the uncorrelated signal information is not used in the DOA estimation process. In other words, we are still estimating $K+K^{2}+1$ parameters.

Next, we reproduce the mean CRB results and include the uncorrelated signal information in the CRB analysis. This seemingly minor change has a significant impact on the mean CRB. Now, the number of unknowns is reduced to 2K+1 because the off-diagonal values of P are known to be zero. This reduces the size of the FIM in the CRB calculations, and as will be shown, significantly impacts the DOA estimation performance of the antenna arrays studied.

**Figure 10:**
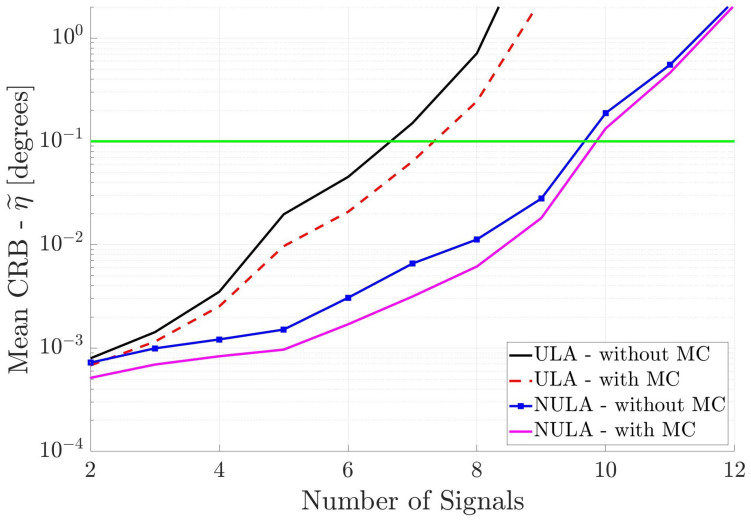
Mean CRB $\left(\tilde{\eta }\right)$ for linear arrays when the incident signals are mutually uncorrelated. This information is used in DOA estimation. The horizontal axis is different from the previous figures.

Figure 10 compares the mean CRB results for the linear arrays when the incident signals are known to be uncorrelated. Note that the horizontal axis in this figure is different than the previous figures. We find that the ULA results are largely unchanged, as the mean CRB curves for the ULA with and without MC crosses the 0.1° threshold before eight incident signals are incident. The NULA has significantly lower mean CRB curves. The NULA with MC has slightly better performance than the NULA without MC, indicating that non-uniform spacing and mutual coupling help improve DOA estimation. Note that now one can estimate the directions of up to ten incident signals. Thus, even small aperture antenna arrays can operate in the underdetermined case (where the number of incident signals is greater than the number of antenna elements).

**Figure 11:**
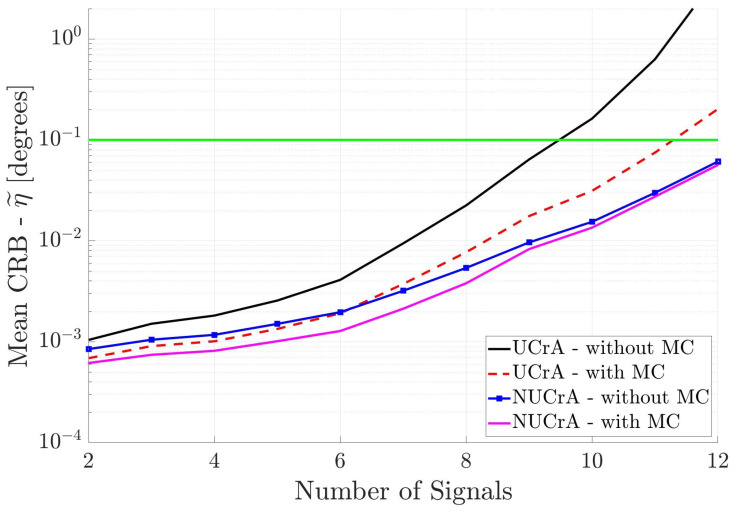
Mean CRB $\left(\tilde{\eta }\right)$ for cross arrays when the incident signals are mutually uncorrelated. This information is used in DOA estimation. The horizontal axis is different from the previous figures.

Figure 11 shows the DOA estimation performance of the two cross arrays when the uncorrelated signal information is used in CRB calculations. All parameters are the same as for Figure 8, except for the uncorrelated incident signal information. The same trend between the arrays is observed; the array with non-uniform spacing and mutual coupling included has the best performance. Also, as compared to the linear antenna arrays in Figure 10, the cross antenna arrays have better DOA estimation performance. Note that with cross antenna arrays one can estimate the directions of more than twelve incident signals. Even the uniform cross array performs better than the uniform linear array (Figure 10) with or without mutual coupling.

**Figure 12:**
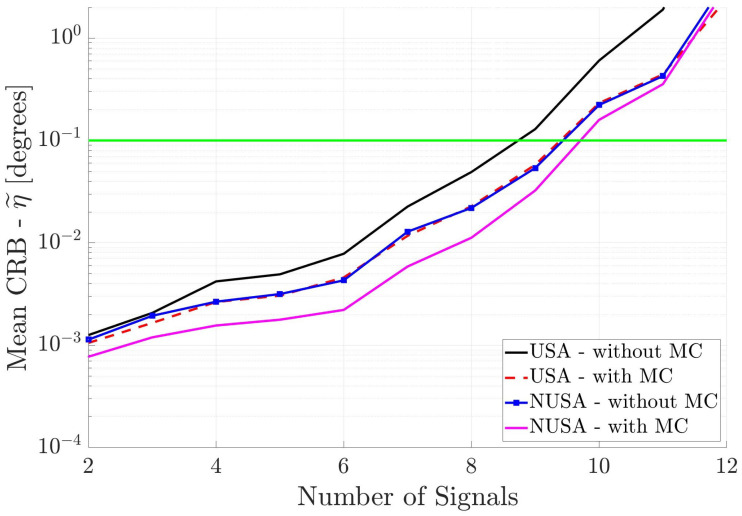
Mean CRB $\left(\tilde{\eta }\right)$ for square arrays when the incident signals are mutually uncorrelated. This information is used in DOA estimation. The horizontal axis is different from the previous figures.

Figure 12 shows the DOA estimation performance of the two square antenna arrays when the incident signals are known to be uncorrelated. All other parameters are the same as in Figure 9. Again, one can see that the non-uniform spacing and inclusion of mutual coupling improves the DOA estimation performance of the antenna array. Square antenna arrays perform better than the linear antenna arrays (Figure 10), but not as well as the cross antenna arrays (Figure 11). The reason for this behavior is that the cross antenna arrays have larger overall aperture than the square antenna arrays.

## 7. Summary and Conclusions

In this paper, we discussed the use of small aperture antenna arrays for DOA estimation. We defined the small aperture antenna array as an array consisting of a few elements with an average interelement spacing of half a wavelength or less. Our analysis showed that arrays with non-uniformly spaced antenna elements should be used for DOA estimation. Furthermore, the DOA estimation performance improved when the antenna array response included MC. Thus, instead of mitigating mutual coupling between antenna elements, it should be measured and included in the DOA estimation process. We applied these observations to linear, cross, and square aperture antenna arrays and demonstrated that non-uniform spacing and the inclusion of mutual coupling leads to significant improvement in DOA estimation as the number of incident signals increases. When all the signals incident on the antenna array are uncorrelated, one can detect more signals than the number of antenna elements in the array. Although the results in this paper used nine element antenna arrays, the ideas and conclusions derived from the results are true for arrays with any number of antenna elements.

## Figures and Tables

**Table 1:** Element distributions of the antenna arrays investigated. All positions are normalized with respect to the wavelength, λ.

	ULA	NULA	UCrA	NUCrA	USA	NUSA
$\left({\bar{x}}_{1},{\bar{y}}_{1}\right)$:	(0,0)	(0,0)	(0,0)	(0,0)	(0,0)	(0,0)
$\left({\bar{x}}_{2},{\bar{y}}_{2}\right)$:	(0.5,0)	(0.1,0)	(−1.0,0)	(−1.0,0)	(0.5,0.5)	(0.5,0.5)
$\left({\bar{x}}_{3},{\bar{y}}_{3}\right)$:	(1.0,0)	(0.4,0)	(−0.5,0)	(−0.3,0)	(0.5,−0.5)	(0.5,−0.5)
$\left({\bar{x}}_{4},{\bar{y}}_{4}\right)$;	(1.5,0)	(1.0,0)	(0.5,0)	(0.4,0)	(−0.5,0.5)	(−0.5,0.5)
$\left({\bar{x}}_{5},{\bar{y}}_{5}\right)$:	(2.0,0)	(1.8,0)	(1.5,0)	(1.0,0)	(−0.5,−0.5)	(−0.5,−0.5)
$\left({\bar{x}}_{6},{\bar{y}}_{6}\right)$:	(2.5,0)	(2.7,0)	(0,−1.0)	(0,−1.0)	(0,0.5)	(−0.4,0.5)
$\left({\bar{x}}_{7},{\bar{y}}_{7}\right)$:	(3.0,0)	(3.3,0)	(0,−0.5)	(0,−0.8)	(0,−0.5)	(0.2,−0.5)
$\left({\bar{x}}_{8},{\bar{y}}_{8}\right)$:	(3.5,0)	(3.8,0)	(0,0.5)	(0,0.4)	(0.5,0)	(0.5,0.3)
$\left({\bar{x}}_{9},{\bar{y}}_{9}\right)$:	(4.0,0)	(4.0,0)	(0,1.0)	(0,1.0)	(−0.5,0)	(−0.5,0.25)

## Data Availability

Data sharing not applicable. All data generated in this paper was done through simulation and can be recreated by following the methodology presented and using the parameters we have provided.
